# Supplementary material for: Memory for nonadjacent dependencies in the first year of life and its relation to sleep
Source: Nat Commun. 2022 Dec 22;13:7896. doi: 10.1038/s41467-022-35558-x (PMC9780241; doi:10.1038/s41467-022-35558-x)
Supplement: Supplementary file 1 — Supplementary Information [file 41467_2022_35558_MOESM1_ESM.pdf]

Memory for nonadjacent dependencies in the first year of life and its relation to sleep

Manuela Friedrich, Matthias Mölle, Jan Born, and Angela D. Friederici

SUPPLEMENTARY INFORMATION

For all supplementary tables and figures, source data are provided as a Source Data file.

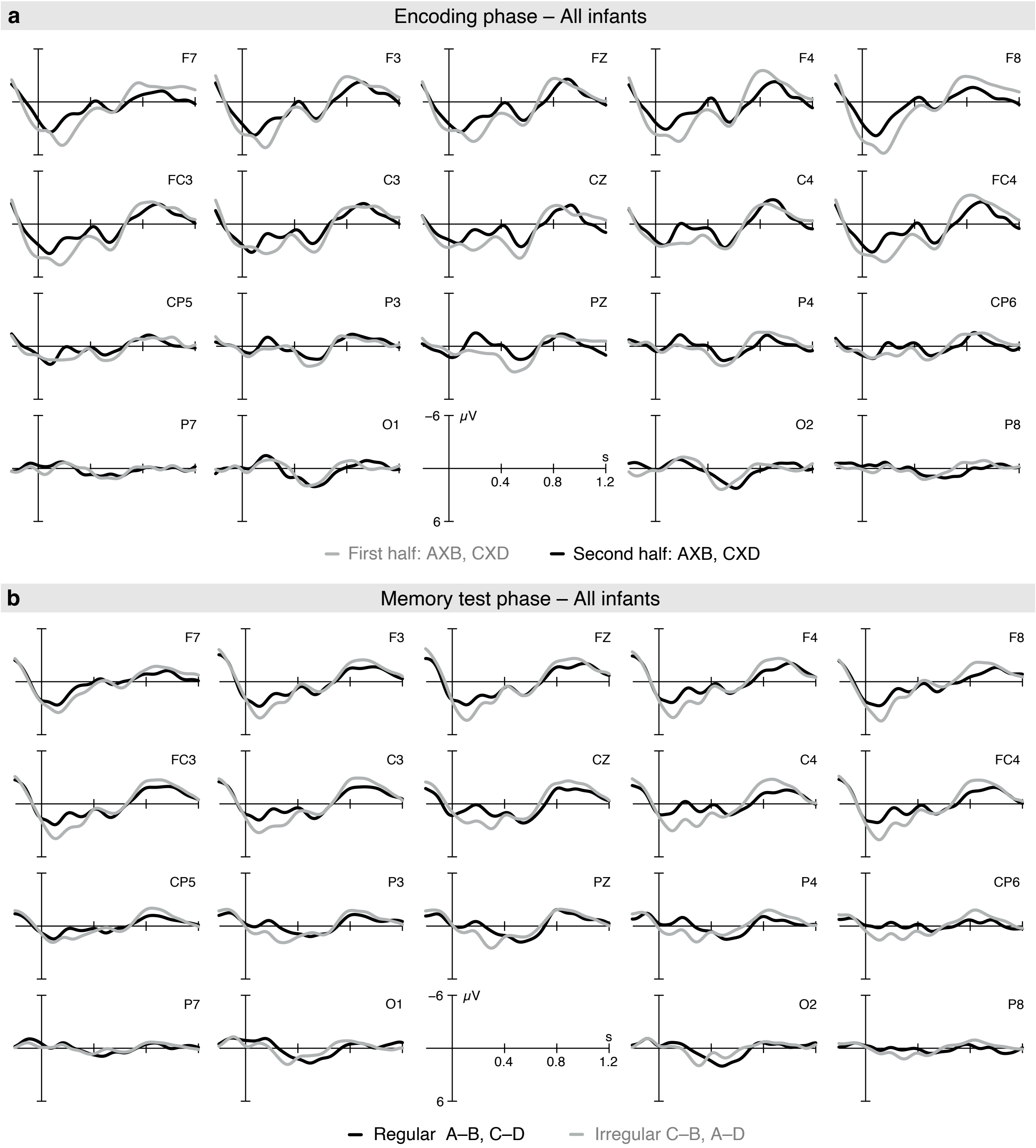

Supplementary Figure 1 Familiarity effects and memory effects.

ERPs at individual electrode positions time-locked to suffix-onset (B and D) in the overall group.

a ERPs for the first half (grey lines) and the second half (black lines) of the encoding phase.

b ERPs for regular (black lines) and irregular (grey lines) sentences in the memory test session.

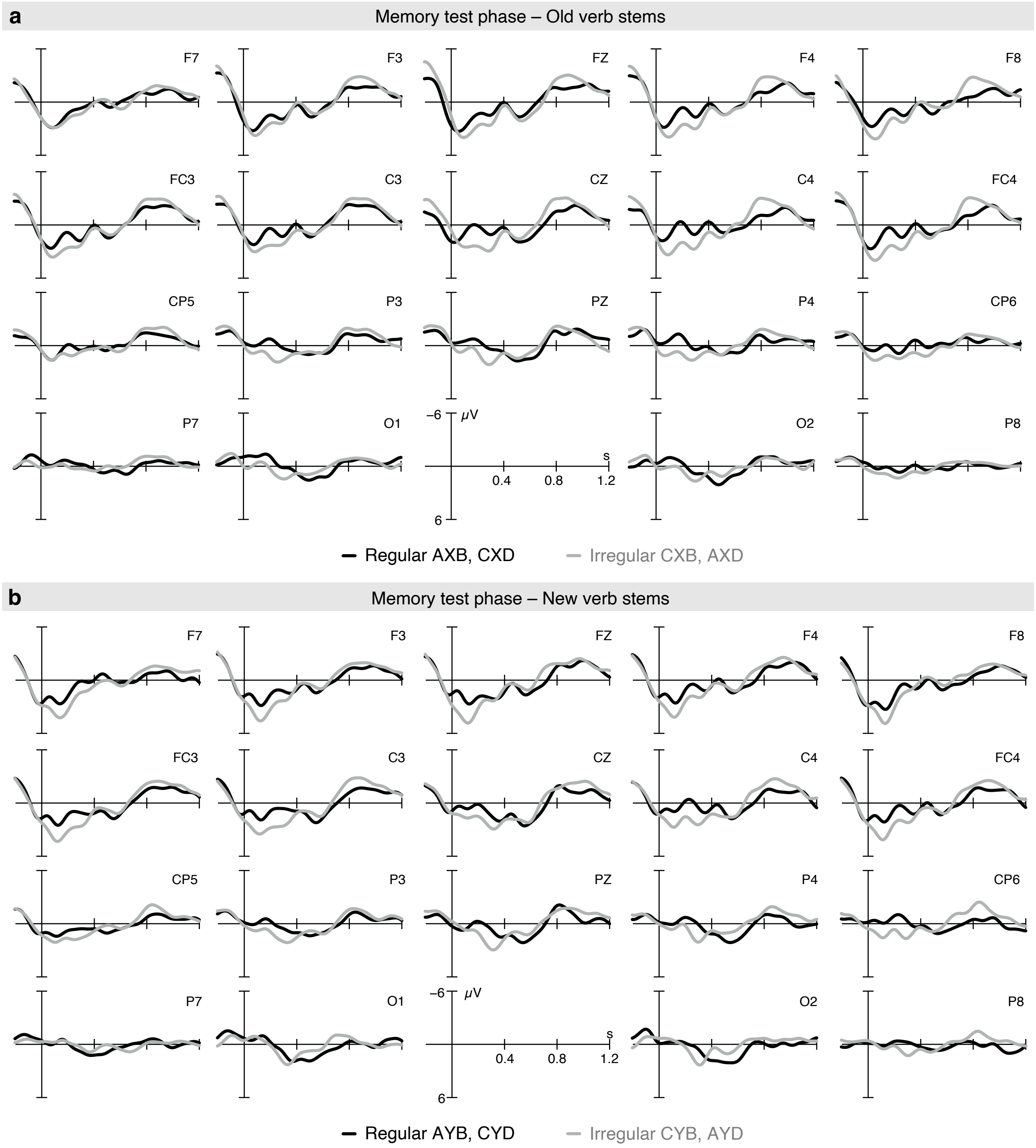

Supplementary Figure 2 Memory effects for old and new verb stems.

ERPs at individual electrode positions for regular (black lines) and irregular (grey lines) sentences time-locked to suffix-onset (B and D) in the overall group.

a ERPs averaged across phrases with old verb stems.

b ERPs averaged across phrases with new verb stems.

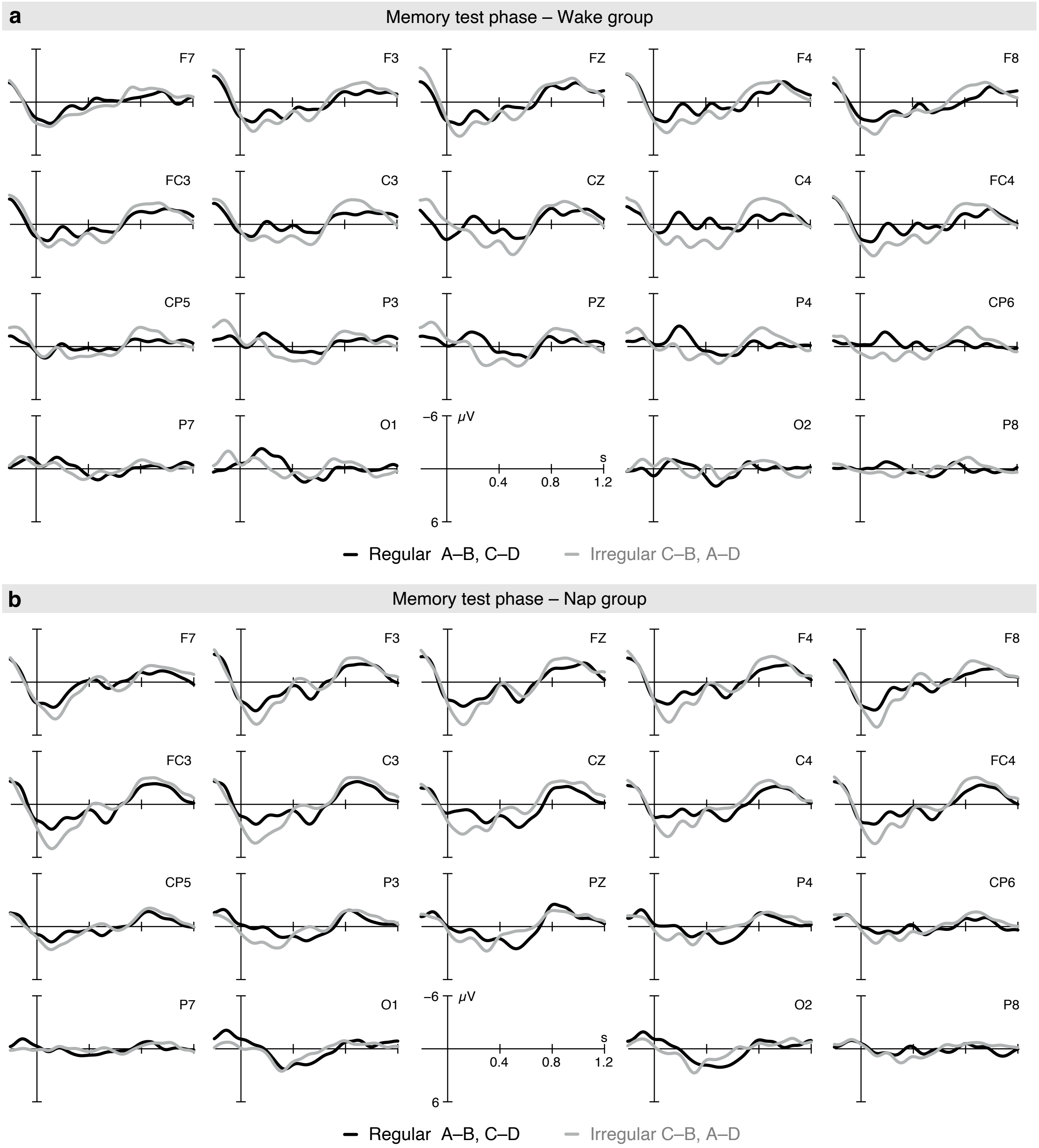

Supplementary Figure 3 Memory effects in the wake and nap groups.

ERPs at individual electrode positions for regular (black lines) and irregular (grey lines) sentences time-locked to suffix-onset (B and D).

a ERPs of the wake group.

b ERPs of the nap group.

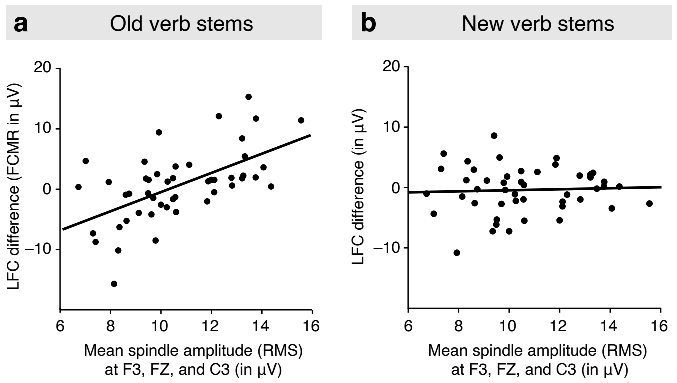

Supplementary Figure 4 Relation between spindle RMS and left frontal-central ERP difference.

a Relation between mean root mean square (RMS) amplitude of sleep spindles measured in left frontal, mid-frontal, and left central regions during the post-encoding nap and the left FCMR (the ERP difference between irregular and regular sentences over the LFC region) for NAD phrases with old verb stems in the memory test phase (Pearson's $r = 0.588$, $P = 0.00001$, 95% CI = [0.365, 0.748], two-sided). The LFC region included the F7, F3, FC3, and C3 positions.

b Lack of relation between mean RMS amplitude of sleep spindles measured in left frontal, mid-frontal, and left central regions and the (non-significant) ERP difference between irregular and regular sentences in the LFC region for phrases with new verb stems (Pearson's $r = 0.049$, $P = 0.740$, 95% CI = [−0.238, 0.329], two-sided). The correlation coefficient for new verb stems significantly differed from that for old verb stems (Meng, Rosenthal, & Rubin's[1,2] $z = -2.748$, $P = 0.006$, two-sided).

Supplementary Table 1 Developmental characteristics of the groups and *t*-tests for group differences (two-sided).

	Nap group		Wake group		*t*-test for independent samples		
	Mean	SD	Mean	SD	*t*	*P*	95% CI
Age in days	220.7	22.33	220.3	26.52	0.087	0.931	[−10.08, 11.00]
Gestational age at birth in days	279.8	8.52	280.1	9.53	−0.147	0.883	[−4.19, 3.61]
Weight at birth in g	3584	491.2	3530	409.8	0.530	0.598	[−147.5, 254.5]
Head circumference at birth in cm*	35.4	1.74	35.4	1.45	0.056	0.955	[−0.73, 0.77]

* Data on head circumference at birth are not included in the Source Data file, because a combination of three or more indirect identifiers could compromise anonymity.[3] (Age is not an indirect identifier here.)

Supplementary Table 2 Sleep characteristics of the nap.

	Mean	SD
Stage 1 sleep in min	9.1	8.49
Stage 2 sleep in min	21.1	10.45
Slow wave sleep in min	9.3	6.37
Total sleep time (TST) in min	39.6	17.84
Spindle peak in Hz	13.7	0.45
Frontal spindle number*	71.1	37.97
Central spindle number*	69.3	37.37
Parietal spindle number*	60.2	35.51
Frontal spindle density in number/30s*	1.2	0.23
Central spindle density in number/30s*	1.2	0.26
Parietal spindle density in number/30s*	1.0	0.31
Frontal spindle peak-to-peak amplitude in µV	42.3	8.85
Central spindle peak-to-peak amplitude in µV	46.3	10.64
Parietal spindle peak-to-peak amplitude in µV	30.8	7.29

The frontal region includes F3, FZ, and F4, the central C3, CZ, and C4, and the parietal P3, PZ, and P4. *during artefact-free periods

Supplementary Table 3 Contribution of left, mid, and right frontal recording sites to the correlation between the amplitude of frontal sleep spindles and the mid-latency N400-like memory effect for old and new verb stems (Person's *r*, two-sided *P*, uncorrected).

Mean ERP amplitude within 500–600 ms	Correlation Pearson's *r*, two-sided *P*	Old verb stems			New verb stems		
		Spindle amplitude at					
		F3	FZ	F4	F3	FZ	F4
Left frontal	*r*	0.375	0.322	0.211	0.036	0.153	0.002
	P	0.009	0.025	0.150	0.805	0.298	0.990
Mid frontal	*r*	0.285	0.282	0.190	0.021	0.121	0.035
	P	0.049	0.052	0.196	0.889	0.413	0.811
Right frontal	*r*	0.178	0.275	0.296	0.183	0.201	0.150
	P	0.227	0.059	0.041	0.212	0.170	0.310
Left central	*r*	0.390	0.371	0.339	0.010	0.228	0.058
	P	0.006	0.009	0.019	0.947	0.118	0.696
Mid central	*r*	0.270	0.331	0.308	0.032	0.201	0.054
	P	0.063	0.022	0.033	0.828	0.171	0.714
Right central	*r*	0.203	0.341	0.324	0.036	0.162	0.007
	P	0.166	0.018	0.025	0.806	0.271	0.963
Left parietal	*r*	0.329	0.447	0.272	0.021	0.218	0.122
	P	0.022	0.001	0.062	0.889	0.137	0.410
Mid parietal-occipital	*r*	0.236	0.364	0.332	– 0.279	– 0.112	0.019
	P	0.107	0.011	0.021	0.055	0.450	0.896
Right parietal	*r*	0.177	0.338	0.196	– 0.101	0.018	– 0.086
	P	0.228	0.019	0.183	0.493	0.901	0.561

Supplementary Table 4 Spindle number and density at individual recording sites in the low- and high-spindle subgroups, *t*-tests for group differences (two-sided *P*, corrected $\alpha = 0.006$).

Sleep spindle parameters		Low-spindle subgroup		High-spindle subgroup		*t*-test for independent samples (corrected α = 0.006, two-sided)			
		Mean	SD	Mean	SD	t_{46}	*P*	*d*	95% CI
Spindle number	F3	56.92	33.08	89.12	39.64	3.056	0.004	0.882	[10.996, 53.421]
	FZ	60.62	34.30	86.33	40.14	2.385	0.021	0.689	[4.014, 47.403]
	F4	55.50	36.41	78.17	33.92	2.232	0.031	0.644	[2.221, 43.112]
	C3	53.79	32.12	80.25	42.67	2.427	0.019	0.701	[4.514, 48.403]
	CZ	61.00	36.94	84.46	39.52	2.125	0.039	0.613	[1.232, 45.684]
	C4	56.46	33.50	79.67	37.28	2.269	0.028	0.655	[2.615, 43.801]
	P3	53.67	30.22	79.12	42.81	2.380	0.022	0.687	[3.929, 46.988]
	PZ	44.58	32.66	60.46	34.43	1.639	0.108	0.473	[–3.625, 35.375]
	P4	52.12	32.29	71.50	37.94	1.905	0.063	0.550	[–1.095, 39.845]
Spindle density	F3	1.10	0.23	1.38	0.25	3.810	0.0004	1.100	[0.125, 0.405]
	FZ	1.18	0.24	1.32	0.21	2.105	0.041	0.608	[0.006, 0.270]
	F4	1.07	0.31	1.21	0.19	2.014	0.050	0.581	[0.000, 0.296]
	C3	1.05	0.35	1.22	0.32	1.704	0.095	0.492	[–0.030, 0.358]
	CZ	1.16	0.37	1.30	0.23	1.604	0.116	0.463	[–0.037, 0.323]
	C4	1.08	0.36	1.24	0.28	1.728	0.091	0.499	[–0.026, 0.344]
	P3	1.03	0.28	1.18	0.37	1.600	0.117	0.462	[–0.039, 0.342]
	PZ	0.82	0.37	0.91	0.35	0.854	0.397	0.247	[–0.121, 0.299]
	P4	0.99	0.32	1.09	0.31	1.084	0.284	0.313	[–0.085, 0.282]

Supplementary References

1. Meng, X.-l., Rosenthal, R., & Rubin, D. B. (1992). Comparing correlated correlation coefficients. *Psychological Bulletin*, *111*(1), 172–175.
2. Hemmerich, W. (2017). StatistikGuru: Korrelationen statistisch vergleichen. Retrieved from https://statistikguru.de/rechner/korrelationen-vergleichen.html
3. Hrynaszkiewicz I., Norton M. L., Vickers A. J., Altman D. G. Preparing raw clinical data for publication: guidance for journal editors, authors, and peer reviewers. Trials. 2010 Jan 29;11:9. doi: 10.1186/1745-6215-11-9.
