# Supplementary material for: Memory for nonadjacent dependencies in the first year of life and its relation to sleep
Source: Nat Commun. 2022 Dec 22;13:7896. doi: 10.1038/s41467-022-35558-x (PMC9780241; doi:10.1038/s41467-022-35558-x)
Supplement: Supplementary file 3 — Reporting Summary [file 41467_2022_35558_MOESM3_ESM.pdf]

nature portfolio

Corresponding author(s): Manuela Friedrich

Last updated by author(s): Nov 19, 2022

Reporting Summary

Nature Portfolio wishes to improve the reproducibility of the work that we publish. This form provides structure for consistency and transparency in reporting. For further information on Nature Portfolio policies, see our Editorial Policies and the Editorial Policy Checklist.

Statistics

For all statistical analyses, confirm that the following items are present in the figure legend, table legend, main text, or Methods section.

n/a	Confirmed	
☐	☒	The exact sample size (*n*) for each experimental group/condition, given as a discrete number and unit of measurement
☐	☒	A statement on whether measurements were taken from distinct samples or whether the same sample was measured repeatedly
☐	☒	The statistical test(s) used AND whether they are one- or two-sided<br>*Only common tests should be described solely by name; describe more complex techniques in the Methods section.*
☒	☐	A description of all covariates tested
☐	☒	A description of any assumptions or corrections, such as tests of normality and adjustment for multiple comparisons
☐	☒	A full description of the statistical parameters including central tendency (e.g. means) or other basic estimates (e.g. regression coefficient) AND variation (e.g. standard deviation) or associated estimates of uncertainty (e.g. confidence intervals)
☐	☒	For null hypothesis testing, the test statistic (e.g. F, t, r) with confidence intervals, effect sizes, degrees of freedom and P value noted<br>*Give P values as exact values whenever suitable.*
☒	☐	For Bayesian analysis, information on the choice of priors and Markov chain Monte Carlo settings
☒	☐	For hierarchical and complex designs, identification of the appropriate level for tests and full reporting of outcomes
☐	☒	Estimates of effect sizes (e.g. Cohen's *d*, Pearson's *r*), indicating how they were calculated

Our web collection on statistics for biologists contains articles on many of the points above.

Software and code

Policy information about availability of computer code

Data collection	- Presentation: Presentation 17.2 (NeuroBehavioral Systems, Berkeley, USA)<br>- EEG data: QREFA 82 (Twente Medical Systems International, Oldenzaal, The Netherlands)<br>- Sleep data: DOMINO Steuerung 2.5 (Somnomedics, Randersacker, Germany)
Data analysis	- EEP 3.2.1. (MPI for Human Cognitive and Brain Sciences, Leipzig), commercially available as EEProbe (ANT Neuro, Germany)<br>- REMBRANDT 9 Diagnostic software / Polysomnography (Natus Medical, Pleasanton, USA)<br>- Spike2 9.12 (Cambridge Electronic Design Limited, Cambridge, United Kingdom)<br>- SPSS Statistics 22 and SPSS 28 (IBM, Armonk, USA)

For manuscripts utilizing custom algorithms or software that are central to the research but not yet described in published literature, software must be made available to editors and reviewers. We strongly encourage code deposition in a community repository (e.g. GitHub). See the Nature Portfolio guidelines for submitting code & software for further information.

Data

Policy information about availability of data

All manuscripts must include a data availability statement. This statement should provide the following information, where applicable:

- Accession codes, unique identifiers, or web links for publicly available datasets
- A description of any restrictions on data availability
- For clinical datasets or third party data, please ensure that the statement adheres to our policy

The data that support the findings of this study have been deposited in the OSF repository and are available at https://osf.io/q2vpg/. Source data are provided with this paper.

Human research participants

Policy information about studies involving human research participants and Sex and Gender in Research.

Reporting on sex and gender	To capture any possible differences between male and female infants, all ANOVAs were repeated with the additional between-subject factor sex (female/male). These analyses yielded the same results and revealed no influence of sex on memory.
Population characteristics	85 monolingual infants growing up in German-speaking families (41 female, mean age 7 months and 7 days, SD 24 days). All infants were born in the 36th to 43st week of pregnancy with a birth weight ranging from 2440 to 4900 g (mean: 3561 ± 456 g). The nap group (N = 48, 25 female) and the wake group (N = 37, 16 female) neither differed in age or birth weight (Supplementary Table 1) nor in gestational age at birth (t83 = −0.147, P = 0.883, 95% CI = [−4.19, 3.61]), head circumference at birth (t74 = 0.056, P = 0.955, 95% CI = [−0.73, 0.77]), or Apgar scores at 10 min after birth (available in 76 of 85 infants, median: 10 in both groups, Mann-Whitney: Z = −0.721, P = 0.471).
Recruitment	Participants were recruited via the local residents' registration office. The self-selection (monolingual, normal hearing, no sleep problems) is not expected to affect the results.
Ethics oversight	All parents gave informed consent before participation. The study complied with all relevant ethical regulations and was approved by the ethics committee of the department of Psychology of the Humboldt University of Berlin.

Note that full information on the approval of the study protocol must also be provided in the manuscript.

Field-specific reporting

Please select the one below that is the best fit for your research. If you are not sure, read the appropriate sections before making your selection.

☒ Life sciences ☐ Behavioural & social sciences ☐ Ecological, evolutionary & environmental sciences

For a reference copy of the document with all sections, see nature.com/documents/nr-reporting-summary-flat.pdf

Life sciences study design

All studies must disclose on these points even when the disclosure is negative.

Sample size	Sample size was determined based on previous infant studies (11,15,22).<br>11. Friedrich et al. Nature Communications 6(1), 1–9 (2015).<br>15. Friedrich et al. Current Biology 27(15), 2374–2380 (2017).<br>22. Friedrich et al. Nature Communications 11(1), 1–9 (2020).
Data exclusions	An additional 40 infants (15 from the nap group, 25 from the wake group) were measured, but not included in the analyses because of: too few artefact-free trials or very noisy ERP responses due to fussiness or too much movement (N = 23), technical problems or loss of data (N = 10), break-off due to strong agitation or crying in one of the experimental sessions (N = 3), failure to fall asleep in the nap group (N = 2), or due to atypical sleep EEG (N = 2).
Replication	The reproducibility of the experimental results is given by the fact that all methods have been described precisely. There are no reasons to assume that the results could not be reproduced. Because this is a novel study, no attempt was made to replicate existing results.
Randomization	Prior to the experimental sessions, infants were assigned to either the wake group or the nap group. Infants of the nap group were scheduled at a time when they were expected to take a nap within 30 min after the encoding session. Infants of the wake group were scheduled at a time when they were expected not to take a nap within the next two to three hours. The group assignment of the consecutively recruited infants was alternating, but changes were sometimes necessary depending on the parents' schedules.

Blinding	The babies were naturally blind to the experimental variation. However, the parents and the experimenter knew about the assignment to the groups, because the babies had to be scheduled according to their typical nap time.

Reporting for specific materials, systems and methods

We require information from authors about some types of materials, experimental systems and methods used in many studies. Here, indicate whether each material, system or method listed is relevant to your study. If you are not sure if a list item applies to your research, read the appropriate section before selecting a response.

Materials & experimental systems

n/a	Involved in the study	
☒	☐	Antibodies
☒	☐	Eukaryotic cell lines
☒	☐	Palaeontology and archaeology
☒	☐	Animals and other organisms
☒	☐	Clinical data
☒	☐	Dual use research of concern

Methods

n/a	Involved in the study	
☒	☐	ChIP-seq
☒	☐	Flow cytometry
☒	☐	MRI-based neuroimaging
